# Supplementary material for: Two Directing Groups Used for Metal Catalysed Meta‐C−H Functionalisation Only Effect Ortho Electrophilic C−H Borylation
Source: European J Org Chem. 2022 Nov 25;2022(44):e202200901. doi: 10.1002/ejoc.202200901 (PMC9828458; doi:10.1002/ejoc.202200901)
Supplement: Supplementary file 1 — Supporting Information [file EJOC-2022-0-s001.pdf]

European Journal of Organic Chemistry

Supporting Information

Two Directing Groups Used for Metal Catalysed *Meta*-C–H Functionalisation Only Effect *Ortho* Electrophilic C–H Borylation

Saqib A. Iqbal[+], Clement R. P. Millet[+], Jürgen Pahl, Kang Yuan, and Michael J. Ingleson*

Two Directing Groups Used for Metal Catalysed *Meta*-C–H Functionalisation Only Effect *Ortho* Electrophilic C–H Borylation

Saqib A. Iqbal+,[a] Clement R. P. Millet+,[a] Jürgen Pahl,[a] Kang Yuan,[a] and Michael J. Ingleson*[a]

[a] Dr. S. A. Iqbal, Dr. C. R. P. Millet, Dr. J. Pahl, K. Yuan, Prof. Dr. M. J. Ingleson
EaStCHEM School of Chemistry, University of Edinburgh
Edinburgh, EH9 3FJ, UK
E-mail: michael.ingleson@ed.ac.uk

[+] These authors contributed equally to this work.

Supporting information for this article is given via a link at the end of the document.

<spi> Part of the joint <url href="https://chemistry-europe.onlinelibrary.wiley.com/doi/toc/10.1002/(ISSN)1099-0690.BoronChemistry">“Boron Chemistry”</url> Special Collection.

Abstract: Two templates used in *meta*-directed C–H functionalisation under metal catalysis do not direct *meta*-C–H borylation under electrophilic borylation conditions. Using BCl_3 only Lewis adduct formation with Lewis basic sites in the template is observed. While combining BBr_3 and the template containing an amide linker only led to amide directed *ortho* C–H borylation, with no pyridyl directed *meta* borylation. The amide directed borylation is selective for the *ortho* borylation of the aniline derived unit in the template, with no *ortho* borylation of the phenylacetyl ring – which would also form a six membered boracycle – observed. In the absence of other aromatics amide directed *ortho* borylation on to phenylacetyl rings can be achieved. The absence of *meta*-borylation using two templates indicates a higher barrier to pyridyl directed *meta* borylation relative to amide directed *ortho* borylation and suggests that bespoke templates for enabling *meta*-directed electrophilic borylation may be required.

Introduction

Directed C–H functionalisation has developed into an extremely powerful methodology to selectively transform arenes. The interaction of the directing group with the catalyst / reagent can overcome the intrinsic reactivity of the arene and enable highly selective C–H functionalisation[1] with regiochemistry otherwise hard to achieve. The *ortho* C–H functionalisation of arenes using covalently bound directing groups is relatively straight forward due to the formation of favoured 5 / 6 membered intermediates / products. In this area C–H borylation is one of the most developed transformations due to the synthetic versatility of C–B containing units.[2] Indeed, a multitude of transition metal catalysed, directed lithiation, and metal free (electrophilic) directed *ortho*-C–H borylation methodologies have been reported.[1a,d,3,4] In contrast, achieving the regioselective *meta* (or *para*) C–H borylation of arenes is much more challenging in the absence of substrate control (such as in the C5 selective iridium catalysed borylation of 1,3-disubstituted aromatics, in contrast the iridium catalysed borylation of mono-substituted aromatics generally leads to mixtures of *meta* and *para* products).[5,6] Nevertheless, notable progress in *meta* and *para* C–H borylation has been reported recently utilising non-covalent interactions.[3b,7] However, these methods require iridium catalysis, thus developing a transition metal-free route, such as electrophilic borylation, to achieve directed *meta* borylation would be highly desirable.

Directed electrophilic C–H borylation proceeds *via* the interaction of a boron Lewis acid (generally BCl_3 or BBr_3) with a covalently bound directing group that contains a sufficiently basic heteroatom.[1d] While the use of covalently bound directing groups to effect *meta* and *para* C–H functionalisation under transition metal (TM) catalysis is now well-precedented,[1e] to the best of our knowledge *meta* C–H borylation has not been reported using the covalently bound directing group approach (with or without TM catalysis). Since the pioneering work of Yu and co-workers,[8] *meta* and *para* C–H functionalisation reactions have been reported using a range of directing groups.[1e,9] Analysis of the covalently attached directing groups successful in *meta* functionalisation show them to be relatively complex due to the requirement to selectively form large rings (e.g. 12 membered) during C–H functionalisation. Therefore, the directing groups generally contain a flexible unit, then one aromatic moiety (or more) and finally a donor atom that's part of a rigid group. The latter is most commonly an Aryl-C≡N unit, however cyano groups are not appropriate as directing groups in electrophilic borylation[3a] due to their low basicity and their tendency to undergo reactions with nucleophiles at C on binding to an electrophile at N (e.g., the Hoesch reaction).

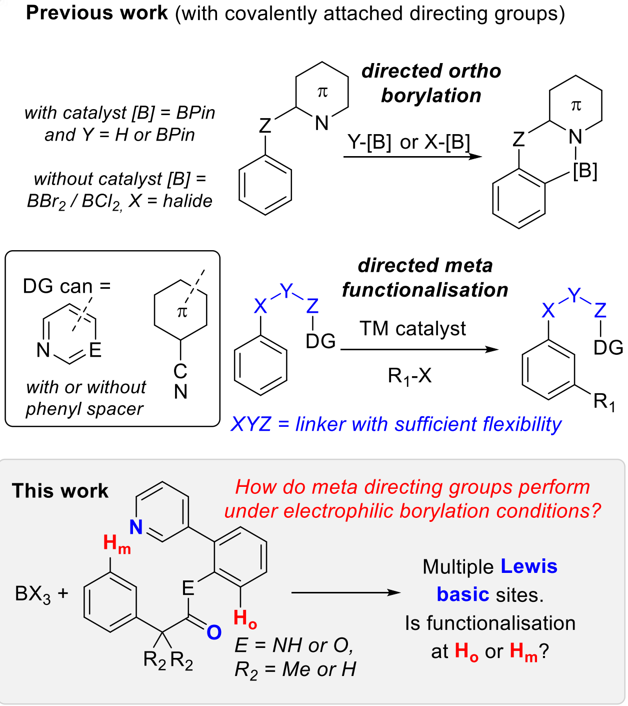

Figure 1. Top, previous work on directed *ortho* C–H borylation. Middle, directed *meta*-C–H functionalisation along with some key features of the templates that enable *meta* selectivity. Bottom, this study using *meta*-C–H functionalisation templates under electrophilic borylation conditions.

Another class of *meta* selective covalently attached directing groups use N-heterocycles as the donor.[10] These include heterocycles such as pyridine and pyrimidine which are well documented to enable directed (*ortho*) electrophilic borylation.[1d,11] However, it should be noted that the *meta* directing groups often contain other basic sites (e.g. amides, imines) in the linker that could also interact with boron electrophiles to effect directed borylation,[12] which would result in the undesired *ortho* borylation. Herein we report our study into how two covalently attached directing groups used in metal catalysed *meta*-C–H functionalisation react under electrophilic borylation conditions.

Results and Discussion

To commence our study, a template that is similar to a directing group pioneered by Yu and co-workers for metal catalysed *meta* functionalisation was selected, compound **1** (Figure 2, top). The major differences between **1** and the successful templates used by Yu (e.g., compound **A**, inset Figure 2) are the absence of a fluorine ortho to N in compound **1**, and the absence of a (R=alkyl) blocking group *ortho* to the aniline NH (used in Yu's report to prevent C–H functionalisation at this position). It was important to avoid the *ortho*-fluorine in **1** as *ortho* halogenated pyridyls have much lower Lewis basicity and are known to bind BX_3 weakly.[13] This is undesirable as it would disfavour BX_3 binding and also make the halide abstraction from the pyridyl→BX_3 derivative more endergonic, this step is essential to form the borenium (three coordinate boron) cation, [pyridyl–BX_2][BX_4] that effects C–H borylation.[14] The *ortho*-to NH alkyl group also was omitted as based on previous studies borylation conditions were envisaged that would only proceed via the borenium cation formed from activation of the pyridyl→BX_3 moiety. Initially BCl_3 was utilised as it is reported that BCl_3 does not affect amide directed electrophilic borylation,[12a,b,15] but it is known to effect pyridyl directed electrophilic borylation.[1d,14a]

Addition of excess BCl_3 to a DCM solution of **1** resulted in a single new species being observed in the *in-situ* ^{1}H NMR spectrum (after 45 mins. at room temperature). However, analysis revealed no C–H borylation (based on integration of the aromatic resonances). Analysis of the ^{11}B NMR spectrum revealed three resonances which were consistent with BCl_3 (δ_{11B}=42.2), the pyridyl→BCl_3 adduct (δ_{11B}=8.4) and the amide(O)→BCl_3 adduct (δ_{11B}=6.8). Leaving the reaction at room temperature or heating it in DCM (to 60 °C in a sealed tube) led to no change in the NMR spectra. The lack of any C–H borylation was confirmed by work-up involving pinacol/NEt_3 addition leading to the formation of no observable C–BPin species by *in-situ* ^{11}B NMR spectroscopy or after work-up. The absence of borylation is in contrast to the reactivity of 2-phenylpyridine and excess BCl_3, which under identical conditions leads to 50% C–H borylation (with the other 50% 2-phenylpyridine protonated by the acidic by-product from S_EAr).[1d] Thus, these findings indicate that the absence of pyridyl directed borylation with **1** is due to a higher barrier to borylation *via* a 12 membered transition state relative to a five membered transition state (in the *ortho* borylation of 2-phenylpyridine). Calculations (see Supporting information, section 12) disfavour substrate electronics precluding pyridyl directed borylation. Specifically, the close in energy HOMO and HOMO-1 of **2** are principally located on the aryl of the $PhCMe_2$- unit and have significant character at the *ortho* and *meta* carbons. Despite this, neither site undergoes pyridyl directed electrophilic borylation under these conditions. The product from addition of excess BCl_3 to **1** was confirmed as the bis-BCl_3 adduct **2** by X-ray crystallography (Figure 2, bottom). The solid-state structure of **2** is unremarkable, containing O–B and N–B bond lengths of 1.485(2) and 1.594(2) Å, respectively, for the Lewis adducts.

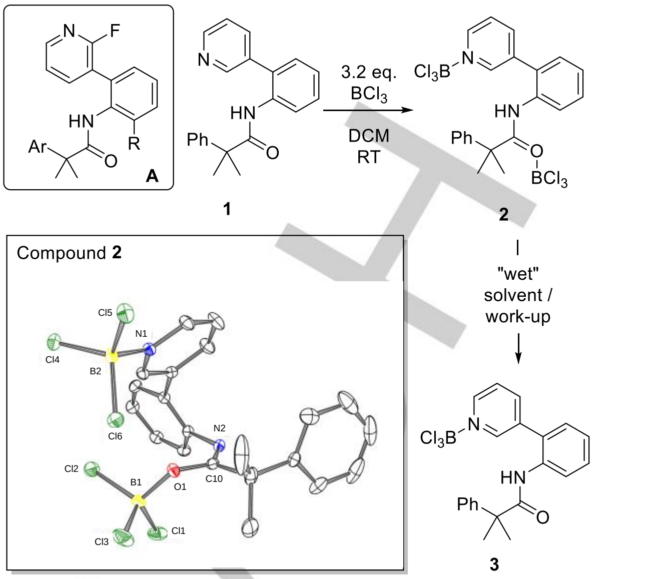

Figure 2. Top left, a template (**A**) successful for metal catalyzed *meta*-C–H functionalisation and it's analogue **1**. Right, formation of **2** and **3**. Inset-bottom, the structure of **2**, ellipsoids at 50% probability. Select distances (Å) and angles (°): O1–B1=1.485(2); N1–B2=1.594(2); C10–O1=1.289(1); Cl3–B1–Cl1=110.59(7); Cl1–B1–Cl2=110.91(7); Cl2–B1–Cl3=109.31(7).

The formation of borenium cations from pyridyl→BCl_3 using additional BCl_3 (to form [pyridyl–BCl_2][BCl_4]) is endergonic,[14a] thus this step will be contributing to the overall barrier to *meta* C–H borylation using **1** / BCl_3. Therefore, the addition of $AlCl_3$ to **2** was explored to make borenium cation formation exergonic and thus lower the overall barrier to *meta* borylation,[16] but this combination failed to affect any C–H borylation (even on heating). Instead, addition of $AlCl_3$ appears to displace BCl_3 from the amide, as only the pyridyl→BCl_3 resonance is observed post $AlCl_3$ addition (at δ_{11B}=8.4 ppm). More insight into the relative stability of the pyridyl-BCl_3 and amide(O)→BCl_3 adducts was forthcoming from the exposure of **2** to "wet" (non-purified) solvent/chromatographic work up which produced compound **3** (Figure 2, right) in which the amide(O)→BCl_3 dative bond had been cleaved, but the pyridyl→BCl_3 bond has persisted (this is indicted by the N-H shifting from δ_{1H}=9.97 for **2** to δ_{1H}=6.62 for **3** and there being only a single δ_{11B} =8.4 now observed for the pyridyl→BCl_3, Figure S1). This confirms the expected stronger binding of BCl_3 to pyridyl relative to amide.

To determine if [pyridyl–BBr_2][BBr_4] boreniums could be accessed selectively (over [amide–BBr_2][BBr_4]) we explored the controlled addition of BBr_3. One equivalent of BBr_3 was added to a DCM solution of **1**. Analysis of the ^{11}B NMR spectrum showed four resonances at δ_{11B}=-1.3, -7.4, -11.5 and -24.3. These resonances can be assigned as follows: the δ_{11B}= -7.4 is in the region expected for pyridyl→BBr_3 adducts, the δ_{11B}= -11.5 is closely comparable to benzoyl→BBr_3 adducts[12a] thus can be assigned to the amide(O)→BBr_3 moiety, the δ_{11B}= -24.3 is consistent with [BBr_4]$^-$, while the broad resonance at δ_{11B}= -1.3 is assigned as the product from amide directed C–H borylation. This indicates that BBr_3 reacts unselectively with the Lewis basic sites in **1** thus can affect amide directed *ortho* borylation even with only 1 equiv. of BBr_3. Indeed, heating the reaction mixture to 60 °C in a sealed tube resulted in disappearance of three of the resonances in the ^{11}B NMR spectrum with only δ_{11B}= -7.4 ppm (pyridyl→BBr_3) persisting and a new broad resonance appearing at δ_{11B}= 0.9 ppm, the latter is in the region for acyl-coordinated aryl–BBr_2 species in 6-membered boracycles.[12a]

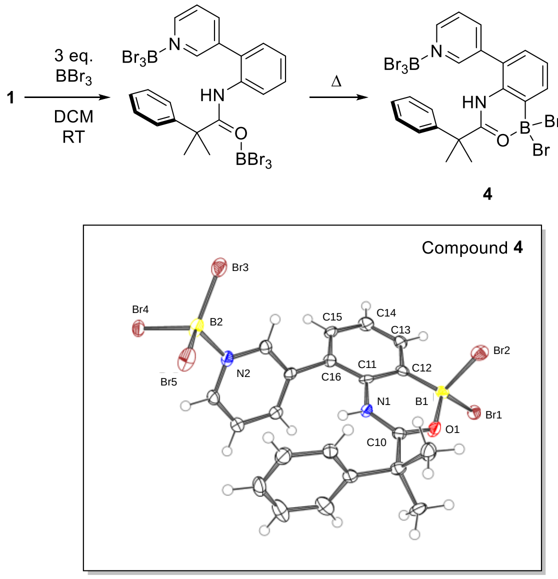

Figure 3. Top, formation of **4**, bottom the structure of **4**, ellipsoids at 50% probability. Distances (Å) and angles (°): N2–B2=1.588(6); O1–B1=1.501(6); O1–C10=1.288(5); Br5–B2–Br4=105.4(3); Br4–B2–Br3=110.7(3); Br3–B2–Br5=114.4(3).

As two boron atoms are incorporated into the product (based on the ^{11}B NMR spectra), >2 equivalents of BBr_3 are required to achieve complete conversion of **1**. Therefore, ca. 3 equivalents of BBr_3 were added to **1**, which led to a complex mixture of species in the ^{1}H NMR spectrum at room temperature. The ^{11}B NMR spectrum exhibited mostly the same resonances as observed when one equivalent of BBr_3 was used, however the resonance at δ_{11B}= -24.3 (due to BBr_4^-) was no longer present and instead a broad resonance at δ_{11B}= +22.0 was observed attributed to a halide transfer equilibrium between BBr_3 and BBr_4^- (*vide infra*). Heating this reaction mixture to 60 °C resulted in complete conversion to a single species in the ^{1}H NMR spectrum that was consistent with compound **4** (Figure 3). Additionally, the *in-situ* ^{11}B NMR spectrum showed the expected three resonances at δ_{11B}= 34.7 (for unreacted BBr_3), 2.9 and -7.8; the δ_{11B}=2.9 resonance is assigned as the C–H borylated unit in species, **4**. Note this species changes chemical shift in the presence of excess BBr_3 due to reversible bromide abstraction (*vide infra*). The species at δ_{11B}= -7.8 is as expected for a pyridyl→BBr_3 moiety. To further confirm this assignment and enable full characterisation crystals suitable for X-ray diffraction analysis were grown by layering a DCM solution of **4** with pentane. The resultant solid-state structure confirmed that *ortho* borylation had occurred *via* amide direction producing a 6-membered boracycle, while the pyridyl group forms a Lewis adduct with BBr_3. *Ortho* borylation occurred exclusively on the aniline derived phenyl and results in planarization of part of the directing template (displacement maximum of 0.023 Å from the plane of O1–B1–C12–C11–N1–C10). Locking the template in the conformation required for the 6-membered boracycle reduces the flexibility of the template and may help prevent *meta* borylation from occurring as post *ortho* borylation, (and using excess BBr_3) prolonged heating of **4** does not lead to any further C–H borylation. This indicates that preventing amide directed *ortho* borylation is essential when using amide containing templates and BBr_3, this is closely related to the findings of Yu with Pd catalysed meta-functionalisation.[10b]

With no *meta* electrophilic borylation observed using substrate **1** due to preferential amide directed *ortho* C–H borylation, the ester analogue, **5**, was next targeted (Scheme 1). Compound **5** was selected as ester / BBr_3 combinations do not affect directed *ortho* borylation[12b] (in contrast to the more basic amide analogues), this will preclude *ortho* borylation without having to install alkyl blocking groups onto the template. Furthermore, an extremely similar ester linked template has been used successfully in palladium catalysed *meta* C–H deuteration,[10c] and alkenyl- / acetoxylation.[17] However, the combination of excess BCl_3 or BBr_3 with compound **5** led to no C–H borylation (*ortho* or *meta*) under a range of conditions, with complex mixtures formed from which the only boron containing species that can be assigned with confidence being due to pyridyl→BX_3 adducts (for X = Cl δ_{11B} = 8.3, for X = Br δ_{11B} = -7.4). The absence of C–H borylation was supported by work up with pinacol/NEt_3, which revealed no species containing C–Bpin moieties were formed (by ^{11}B NMR spectroscopy). Therefore, the failure of template **5** in *meta* borylation under standard electrophilic borylation conditions is not due to preferential *ortho* borylation but is presumably due to a high energy barrier to electrophilic borylation using BX_3 *via* a 12 membered transition state.

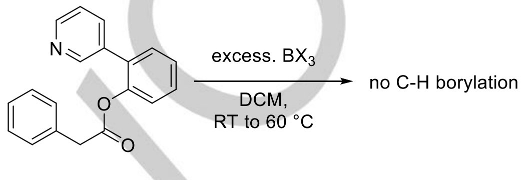

5

Scheme 1. Attempted borylation of compound **5** with BX_3 (X = Cl or Br).

It is notable that during the amide directed borylation of **1** only one *ortho* borylation product is formed, compound **4**, with no alternative *ortho* borylation product, compound **B**, observed (Figure 4), despite the presence of the CMe_2 unit in the linker which may have been expected to favour ring closure onto the phenylacetyl unit. Therefore, we were interested in the feasibility of amide directed C–H borylation where a six membered boracycle is still formed but there is one sp^3 unit in the linker (e.g. the CH_2 and CMe_2 groups in **6** and **7**). To the best to our knowledge amide directed electrophilic borylation has not been reported to date for these types of substrates.

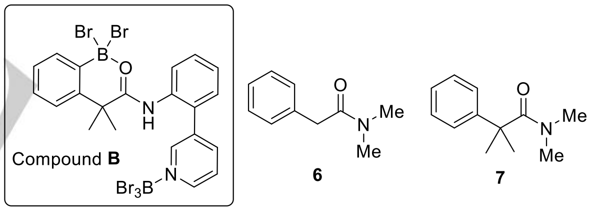

Figure 4. Inset, the unobserved *ortho* borylation isomer, **B**. Right, compounds **6** and **7** used to assess viability of carbonyl directed electrophilic borylation.

The reaction of **6** with 2.5 equivalents of BBr_3 resulted in a species containing a resonance in the ^{11}B NMR spectrum at δ_{11B}= -11.0, in the region for an amide(O)→BBr_3 adduct, thus it is tentatively assigned as **6-BBr$_3$** (Figure 5, top). Heating of the reaction mixture to 60 °C is required to result in the formation of a C-H borylated species. This contains only four aromatic protons (Figure S2) and a resonance consistent with HBr formation (δ_{1H}=-2.6 ppm observed only pre-vacuum treatment) – the by-product of C–H borylation (visible only in these sealed tube conditions). However, two new resonances were observed in the *in-situ* ^{11}B NMR spectrum suggesting two boron centers are incorporated into the product. Under these conditions the two new resonances in the *in-situ* ^{11}B NMR spectrum are at δ_{11B}= +10.2 and +42.8, which we assign as an equilibrium between **8A** and **8B**, with the BBr_3 associated with either O or N in **8B** as it is not removed *in-vacuo*. Note the ^{11}B chemical shifts were dramatically affected by the equivalents of BBr_3 used in this reaction (*vide infra*).

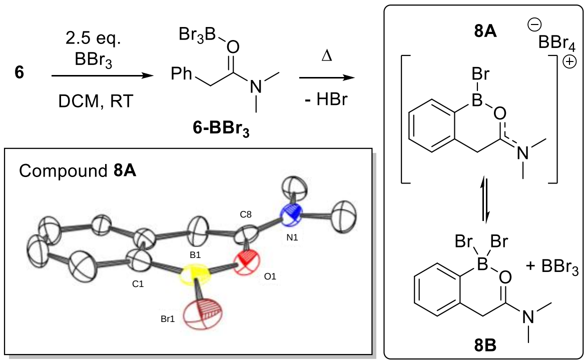

Figure 5. Borylation of compound **6** with BBr_3. Bottom, the solid-state structure of the cationic portion of compound **8A**. Anion not shown for clarity**.** Ellipsoids at the 50% probability level. Selected distances (Å) and angles (°): shortest Br_{anion}–$B1_{cation}$=3.474(6); Br1–B1=1.902(6); B1–O1=1.384(7); O1–C8=1.336(6); C1–B1–Br1=124.3(4); Br1–B1–O1=114.4(4); O1–B1–C1=121.3(5).

Crystals of the borylated product suitable for X-ray diffraction studies were grown by slow diffusion of pentane into a DCM solution of **8A**/**8B**. The solid-state structure (Figure 5, bottom) confirmed the presence of two boron molecules in the product, as in **8A**, in the form of a cation and a $[BBr_4]^-$ counteranion. In the structure of **8A** the closest B···Br–BBr_3 contact is at 3.474(6) Å, within the combined van der Waals radii for B and Br (Σ = 3.75 Å)[18] suggesting transfer of bromide between cation and anion is possible in solution (*vide infra* for more detailed structural discussion). To further examine the proposed equilibrium between **8A** and **8B**, variable temperature NMR spectroscopy studies were conducted. Incremental cooling of a DCM solution of **8A/8B** (Figure 6, made from **6** and 2.5 equiv. of BBr_3 and heated and then dried *in-vacuo*) showed a gradual change in the ^{11}B NMR spectra whereby the boron centre in the cationic component was shifted gradually downfield from δ_{11B}= 37.5 to δ_{11B} ca. 42 ppm (in the range expected for an ArylB(OR)Br species)[19], whereas the second resonance was shifted upfield from δ_{11B}= -16.6 to δ_{11B}= -25 ppm (Figure 6), the latter is as expected for a discrete BBr_4^- anion. Thus, it can be concluded that upon cooling of the sample, the product favours the salt form, **8A**. We also conducted studies involving the addition of increasing amounts of excess BBr_3 to the **8A/8B** mixture (from 0 to 6 equiv.) to probe the effect on both ^{11}B resonances (Figure S5); while the cationic component moves to a limiting δ_{11B}= +43.2 (consistent with the three coordinate boron centre in **8A**), the second resonance shifts closer and closer to that for free BBr_3 (e.g. at 0 equiv. excess BBr_3 δ_{11B} = -10.2 and at 6 additional equivalents of BBr_3 δ_{11B} = 30.1 ppm), as expected for a fast exchange of bromide between BBr_3/BBr_4 and an increasing quantity of BBr_3.

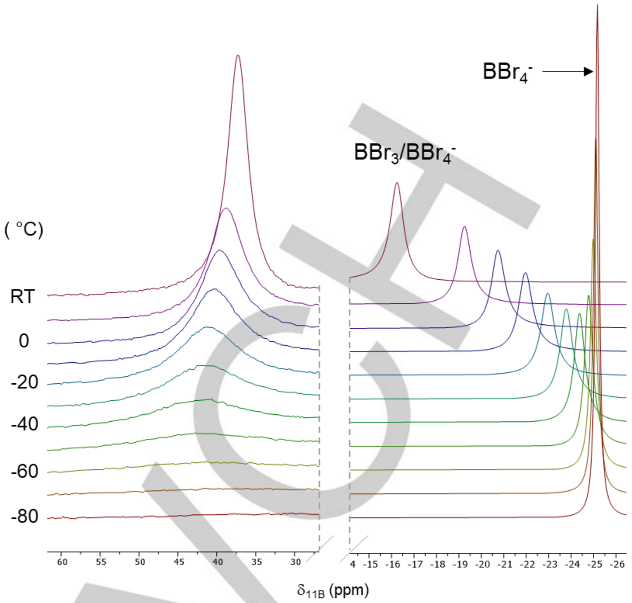

Figure 6. Variable (10 °C increments) temperature ^{11}B NMR spectra for the product **8A/8B.** Measured on a 142 mM DCM solution of **8A/8B**.

With an understanding of the products formed from **6**/ BBr_3 compound **7** was reacted with BBr_3. While the Lewis adduct **7-BBr₃** forms rapidly, using 2.5 equivalents of BBr_3 and heating at 60 °C (in a sealed vessel) resulted in incomplete conversion of **7-BBr₃** to the borylated species **9A**/**9B**, after 16 hours. High levels of conversion (by NMR spectroscopy) to **9A**/**9B** required 3 days of heating. It should be noted only **9A** is shown in Figure 7, but an analogous equilibrium occurs for **9A** (to form **9B**) as observed for **8A** / **8B**. It is noteworthy that the borylation of **7** is slower than the borylation of **6**, this is attributed to steric clash between the –NMe_2 moiety and the –CMe_2 in **7-BBr₃** (and the borenium derived from **7-BBr₃**). Such interactions will presumably lead to rotation around the Me_2C–C(O)NMe_2 bond to orientate the NMe_2 unit to reduce clash with the CMe_2 unit and thus position the boron centre unfavourably for C–H borylation (disfavouring formation of the key transition state -which itself maybe higher in energy due to the unfavourable interactions between NMe_2/CMe_2 when the carbonyl is positioned appropriately). Regardless, the formation of **8A/8B** and **9A/9B** confirms that carbonyl directed electrophilic borylation tolerates CH_2 / CMe_2 groups in the linker. Therefore the preference to form compound **4** over **B** is attributed to a lower kinetic barrier to borylate the aniline derived unit in carbonyl directed borylation (which has been previously observed to undergo borylation at room temperature).[12a,b,15b] As noted earlier, borylation selectivity is not controlled by the location of the HOMO/HOMO-1 in this case, as both these orbitals are principally located on the aryl unit of ArylCMe_2. Despite this borylation still proceeds on the aniline derived unit to form **4**.

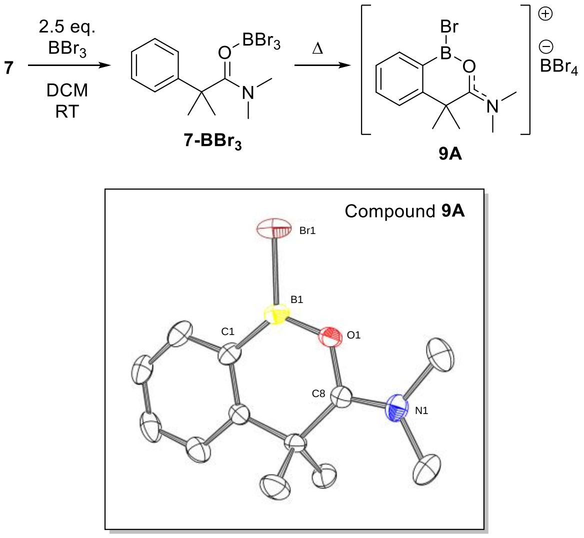

Figure 7. Top borylation of **7**, (note **9A** exists in equilibrium with **9B**, not shown, but is analogous to that discussed for **8A**/**8B)**. Bottom, solid-state structure of **9A** with BBr_4^- anion omitted for clarity. Ellipsoids at the 50% probability level. Selected distances (Å) and angles (°): shortest Br_{anion}–$B1_{cation}$=3.487(5); O1–C8=1.336(6); O1–B1=1.378(6); Br1-B1-O1=113.8(4); O1–B1–C1=120.8(4); C1–B1–Br1=125.4(4).

The structure of **9A** was confirmed by X-ray diffraction studies (Figure 7 bottom). In the solid state, both compounds **8A** and **9A** show short BBr_4···B_{cation} contacts between a bromide and boron of 3.474(6) and 3.487(5) Å for **8A** and **9A,** respectively. Notably, due to the planar N1–C8–O1–B1 unit **9A** has one of the methyls of the –NMe_2 orientated between the two CMe_2. Thus, the structure of **9A** shows minimal deviation of planarity between the plane of the cyclic boronate ring and the –NMe_2 (max. 0.026 Å), whereas the –NMe_2 moiety in **8A** is deviated by upto 0.423 Å from the plane of the boracycle. Here, the entire –NMe_2 unit in **8A** is bent out of the plane possibly due to packing effects (as short contacts of 2.92–2.98 Å are observed between the –N(C**H**$_3$)$_2$ and BBr_4^- anion) of the cyclic boronate framework with the C–NMe_2 moiety remaining planar (angles around N Σ = 359.9° for both **8A** and **9A**). Additionally, the angles around C8 in both **8A** and **9A** sum to to 359.9 and 360.0°, respectively. Other notable features include the C–O bond in the cations which at 1.336(6) Å is lengthened relative to an uncoordinated amide C=O bond (typically ~1.23 Å in length).[20] This is as expected for a carbonyl unit upon Lewis acid coordination.[21] Additionally, the N1–C8 bond length is 1.287(7) and 1.291(7) Å for **8A** and **9A,** respectively, slightly shortened relative to that in an uncoordinated amide (typically ~1.35 Å).[20] Thus, the slightly contracted C–N and lengthened C=O suggest delocalisation of the cationic charge across the B–O–C–N unit, and thus the cationic products **8A** and **9A** can be considered to have iminium character as well borocation character (i.e. the positive charge in these cations will be localised predominantly on the least electronegative atoms, in this case C and B, as previously observed in other borenium cations).[22] Next, the conversion of **8A**/**9A** into bench stable products familiar to synthetic chemists was targeted. Attempts to form the pinacol-protected product derived from **8A** was successful using pinacol and NEt_3, with the product having a δ_{11B}= 30.7 ppm. However, in our hands we could not isolate this product sufficiently pure due to its instability on silica. Isolation of the pure *ortho* borylated products as bench stable compounds was achieved by protecting at boron with 1,8-diaminonaphthalene (1,8-Dan) to form –BDan protected **10** and **11** in 65% and 34% isolated yields, respectively, *via* a one-pot procedure (Scheme 2). The ^{11}B NMR spectra of the –BDan protected compounds each showed a single resonance at δ_{11B}= 30–31, consistent with a 3-coordinate boron centre. The absence of any significant B–O dative bond post Dan installation is similar to the ^{11}B NMR spectra obtained for the pivaloyl-directed borylation of anilines which show minimal coordination to the carbonyl directing group after pinacol installation.[12a]

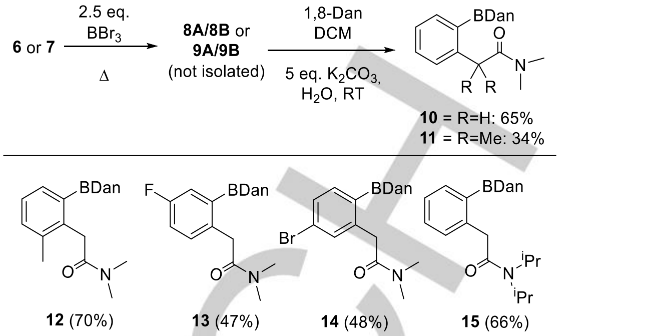

Scheme 2. Top, formation of BDan boronates, **10** / **11** by directed *ortho* borylation. Bottom, further BDan substrates formed by amide directed C-H borylation.

Finally, several additional substrates related to **6** were explored to test the generality of this directed electrophilic borylation process. The successful formation of compounds **12-14** (Scheme 2, bottom) demonstrates that substituents in the *o*, *m* and *p* positions are tolerated. While the C-H borylation step in the formation of **12** occurs under the same conditions as that for **6**, having a fluoride *meta* to the C-H borylation site significantly retards the electrophilic borylation step. For this substrate borylation required 24 h at 100ºC (in chlorobenzene) to produce reasonable yields of **13** post protection. The *meta* (to the acetylamide unit) bromo derivative leads to two inequivalent *ortho* C-H positions, but borylation is only observed at the less hindered *ortho* site. Again, the electrophilic borylation step is slower relative to that of **6**, this time due to the electron withdrawing bromo group *para* to the C-H borylation position. While requiring longer reaction times/higher temperatures (than **6**), the successful formation of **13** and **14** nevertheless demonstrates that challenging substrates (in terms of substrates that are deactivated towards S_EAr) can be borylated with high selectivity and reasonable yield using this methodology. Finally, variation in the nitrogen substituents was investigated, with bulkier ^{i}Pr substituents used in place of methyl. This led to formation of **15** in reasonable yield, with the C-H borylation step proceeding to high conversion within 24 h at 60ºC (by-*in-situ* NMR spectroscopy and by isolation of the intermediate before protection with 1,8-Dan).

Conclusion

In summary, two close analogues of directing templates effective in transition metal catalysed *meta*-C–H functionalisation were not able to effect *meta* directed C–H borylation *via* electrophilic borylation under a range of conditions. Thus bespoke (i.e., not transferred directly from transition metal catalysed approaches) covalently attached directed groups may be required to enable *meta*-selective electrophilic borylation. Removing any Lewis basic groups in the template that could affect *ortho* C-H borylation (via 5 or 6 membered boracycles) is one obvious next step emerging from this work. Indeed, the template with an amide unit can be used to effect amide directed *ortho* borylation which proceeds selectively on the aniline derived aryl unit in preference to the phenylacetyl unit. Phenylacetyl units were shown to be amenable to carbonyl directed *ortho* borylation with BBr_3on heating. This process tolerated electron withdrawing substituents and groups at all three positions on the aryl unit.

Acknowledgements

This project has received funding from the European Research Council (ERC) under the European Union's Horizon 2020 research and innovation programme (grant agreement No 769599). We thank the Mass Spectrometry facility (SIRCAMS) at the University of Edinburgh for carrying out MS analysis. We thank Dr Lorna Murray from the NMR facility at the University of Edinburgh for assistance with variable temperature NMR

spectroscopy. We also acknowledge Dr Gary Nichol from the X-ray crystallography department at the University of Edinburgh for collecting single crystal data.

Conflict of Interest

The authors declare no conflict of interest.

Keywords: Boron • Directing groups • Electrophilic substitution • Meta-C-H Functionalisation

[1] a) A. Ros, R. Fernández, J. M. Lassaletta, *Chem. Soc. Rev.*, **2014**, 43, 3229–3243. b) S. Rej, Y. Ano, N. Chatani, *Chem. Rev.*, **2020**, 120, 1788–1887. c) M. Zhang, Y. Zhang, X. Jie, H. Zhao, G. Li, W. Su, *Org. Chem. Front.*, **2014**, 1, 843–895. d) S. A. Iqbal, J. Pahl, K. Yuan, M. J. Ingleson, *Chem. Soc. Rev.*, **2020**, 49, 4564–4591. e) A. Dey, S. K. Sinha, T. K. Achar, D. Maiti, *Angew. Chem. Int. Ed.*, **2019**, 58, 10820–10843.

[2] *Boronic Acids: Preparation and Applications*, D. Hall, Wiley-VCH, Weinheim, **2011**.

[3] a) S. Kirschner, K. Yuan, M. J. Ingleson, *New J. Chem.*, **2021**, 45, 14855–14868. b) R. Bisht, C. Haldar, M. M. M. Hassan, M. E. Hoque, J. Chaturvedi, B. Chattopadhyay, *Chem. Soc. Rev.*, **2022**, 51, 5042–5100. C) S. Hazra, S. Mahato, K. K. Das, S. Panda, *Chem. Eur. J.* **2022**, ASAP, e202200556

[4] T. E. Hurst, T. K. Macklin, M. Becker, E. Hartmann, W. Kügel, J. C. Parisienne-LaSalle, A. S. Batsanov, T. B. Marder, V. Snieckus, *Chem. Eur. J.*, **2010**, 16, 8155–8161.

[5] For a review on C–H activation to form C–B bonds, see: I. A. I. Mkhalid, J. H. Barnard, T. B. Marder, J. M. Murphy, J. F. Hartwig, *Chem. Rev.*, **2010**, 110, 890–931.

[6] a) J. Y. Cho, M. K. Tse, D. Holmes, R. E. Maleczka, M. R. Smith, *Science*, **2002**, 295, 305–308. b) T. Ishiyama, J. Takagi, J. F. Hartwig, N. Miyaura, *Science*, **2002**, 41, 3056–3058.

[7] M. T. Mihai, G. R. Genov, R. J. Phipps, *Chem. Soc. Rev.*, **2018**, 47, 149–171.

[8] D. Leow, G. Li, T. S. Mei, J. Q. Yu, *Nature*, **2012**, 486, 518–522.

[9] J. Yang, *Org. Biomol. Chem.*, **2015**, 13, 1930–1941.

[10] a) A. Gholap, S. Bag, S. Pradhan, A. R. Kapdi, D. Maiti, *ACS Catal.*, **2020**, 10, 5347–5352. b) Z. Jin, L. Chu, Y. Q. Chen, J. Q. Yu, *Org. Lett.*, **2018**, 20, 425–428. c) H. Xu, M. Liu, L. J. Li, Y. F. Cao, J. Q. Yu, H. X. Dai, *Org. Lett.*, **2019**, 21, 4887–4891.

[11] For select examples of directed electrophilic C-H borylation using N donors published since 1d see: a) M. Vanga, A. Sahoo, R. A. Lalancette, *Angew. Chem. Int. Ed.* **2022**, 61, e202113075. b) G. Wu, B. Pang, Y. Wang, L. Yan, L. Chen, T. Ma, Y. Ji, *J. Org. Chem.* **2021**, 86, 5933-5942. c) M. Shigeno, M. Imamatsu, Y. Kai, M. Kiriyama, S. Ishida, K. Nozawa-Kumada, Y. Kondo, *Org. Lett.* **2021**, 23, 8023-8027. d) G. Wu, X. Fu, Y. Wang, K. Deng, L. Zhang, T. Ma, Y. Ji, *Org. Lett.* **2020**, 22, 7003-7007. e) S. Rej, A. Das, N. Chatani, *Chem. Sci.* **2021**, 12, 11447-11454. f) G. Wu, Z. Yang, X. Xu, L. Hao, L. Chen. Y. Wang, Y. Ji, *Org. Lett.* **2022**, 24, 3570-3575.

[12] a) S. A. Iqbal, J. Cid, R. J. Procter, M. Uzelac, K. Yuan, M. J. Ingleson, *Angew. Chem. Int. Ed.*, **2019**, 58, 15381–15385. b) J. Lv, X. Chen, X.-S. Xue, B. Zhao, Y. Liang, M. Wang, L. Jin, Y. Yuan, Y. Han, Y. Zhao, Y. Lu, J. Zhao, W.-Y. Sun, K. N. Houk, Z. Shi, *Nature*, **2019**, 575, 336–340. c) S. Rej, N. Chatani, *J. Am. Chem. Soc.*, **2021**, 143, 2920–2929. d) Z.-J. Wang, X. Chen, L. Wu, J. J. Wong, Y. Liang, Y. Zhao, K. N. Houk, Z. Shi, *Angew. Chem. Int. Ed.* **2021**, 60, 8500-8504.

[13] A. Del Grosso, J. Ayuso Carrillo, M. J. Ingleson, *Chem. Commun.*, **2015**, 51, 2878–2881.

[14] a) S. A. Iqbal, K. Yuan, J. Cid, J. Pahl and M. J. Ingleson, *Org. Biomol. Chem.*, **2021**, 19, 2949–2958. b) D. Y. Wang, H. Minami, C. Wang and M. Uchiyama, *Chem. Lett.*, **2015**, 44, 1380–1382. c) N. Ishida, T. Moria, T. Goya, M. Murakami, *J. Org. Chem.* **2010**, 75, 8709-8712.

[15] a) In this study and in our prior studies (ref. 12a) on amide directed borylation with BCl_3, no C–H borylated products were observed. Minor conversion to C–H borylation products was observed in one other example of amide directed borylation with BCl_3 but it was at ca. 5% yield, see: b) J. Lv, B. Zhao, Y. Yuan, Y. Han, Z. Shi, *Nat. Commun.*, **2020**, 11, 1316.

[16] a) G. E. Ryschkewitsch, J. W. Wiggins, **1970**, 92, 1790–1791. b) M. J. Ingleson, *Synlett*, **2012**, 23, 1411–1415. c) V. Bagutski, A. Del Grosso, J. Ayuso Carrillo, I. A. Cade, M. D. Helm, J. R. Lawson, P. J. Singleton, S. A. Solomon, T. Marcelli, M. J. Ingleson, *J. Am. Chem. Soc.*, **2013**, 135, 474-487.

[17] B. Jiao, Z. Peng, Z. H. Dai, L. Li, H. Wang, M. D. Zhou, *Eur. J. Org. Chem.*, **2019**, 3195–3202.

[18] M. Mantina, A. C. Chamberlin, R. Valero, C. J. Cramer, D. G. Truhlar, *J. Phys. Chem. A*, **2009**, 113, 5806–5812.

[19] M. C. Haberecht, M. Bolte, H. W. Lerner, M. Wagner, *Eur. J. Inorg. Chem.*, **2005**, 4309–4316.

[20] E. Wei, B. Liu, S. Lin, F. Ling, *Org. Biomol. Chem.,* **2014**, 12, 6389–6392.

[21] D. J. Parks, W. E. Piers, M. Parvez, R. Atencio, M. J. Zaworotko, *Organometallics*, **1998**, 17, 1369–1377.

[22] a) S. A. Solomon, A. Del Grosso, E. R. Clark, V. Bagutski, J. J. W. McDouall, M. J. Ingleson, *Organometallics*, **2012**, 31, 1908–1916. b) M. A. Dureen, A. Lough, T. M. Gilbert, D. W. Stephan, *Chem. Commun.*, **2008**, 913, 4303–4305.

[23] <url href="https://www.ccdc.cam.ac.uk/services/structures?id=doi:10.1002/ejoc.202200901">Deposition Numbers 2192730 (for **2**), 2192729 (for **4**), 2192731 (for **8A**), and 219272932 (for **9A**)</url> contain the supplementary crystallographic data for this paper. These data are provided free of charge by the joint Cambridge Crystallographic Data Centre and Fachinformationszentrum Karlsruhe Access Structures service <url href="https://www.ccdc.cam.ac.uk/structures/?">www.ccdc.cam.ac.uk/structures</url>.

Entry for the Table of Contents

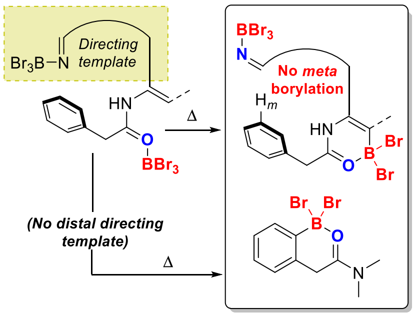

Two templates used in metal catalysed directed meta functionalisation led to either no borylation, or only ortho borylation under electrophilic borylation conditions. In the absence of the complex template amide directed ortho borylation onto phenylacetyl groups is shown to be possible.

Twitter:
@InglesonMike

WILEY-VCH
